# Loss of Endocan tumorigenic properties after alternative splicing of exon 2

**DOI:** 10.1186/1471-2407-8-14

**Published:** 2008-01-18

**Authors:** Florence Depontieu, Bogdan-Dragos Grigoriu, Arnaud Scherpereel, Estelle Adam, Maryse Delehedde, Philippe Gosset, Philippe Lassalle

**Affiliations:** 1 INSERM U774, Lille 59019, France; 2 Pasteur Institute, Lille 59019, France; 3 University of Medicine and Pharmacy, Iasi 700111, Romania; 4 Clinique des Maladies Respiratoires, Hôpital A Calmette, CHRU, Lille 59037, France

## Abstract

**Background:**

Endocan was originally described as a dermatan sulfate proteoglycan found freely circulating in the blood. Endocan expression confers tumorigenic properties to epithelial cell lines or accelerate the growth of already tumorigenic cells. This molecule is the product of a single gene composed of 3 exons. Previous data showed that endocan mRNA is subject to alternative splicing with possible generation of two protein products. In the present study we identified, and functionally characterized, the alternative spliced product of the endocan gene: the exon 2-deleted endocan, called endocanΔ2.

**Methods:**

Stable, endocanΔ2-overexpressing cell lines were generated to investigate the biological activities of this new alternatively spliced product of endocan gene. Tumorigenesis was studied by inoculating endocan and endocanΔ2 expressing cell lines subcutaneously in SCID mice. Biochemical properties of endocan and endocanΔ2 were studied after production of recombinant proteins in various cell lines of human and murine origin.

**Results:**

Our results showed that the exon 2 deletion impairs synthesis of the glycan chain, known to be involved in the pro-tumoral effect of endocan. EndocanΔ2 did not promote tumor formation by 293 cells implanted in the skin of severe combined immunodeficient (SCID) mice.

**Conclusion:**

Our results emphasize the key role of the polypeptide sequence encoded by the exon 2 of endocan gene in tumorigenesis, and suggest that this sequence could be a target for future therapies against cancer.

## Background

Tumor development is a complex multi-step mechanism, involving not only the tumor cells but also the microenvironment (or the stroma) supporting them [1]. Crucial efforts were done to identify the key molecular players that orchestrate the tumor – stromal cell interactions, the neo-blood vessel recruitment and extracellular matrix organization [2-4]. The proteoglycans are major constituents of the tumor stroma and the vascular bed and are present at the cell surface and in the extracellular matrix [1]. They are constituted of a protein core with one or more covalently attached glycosaminoglycan (GAG) chains. Proteoglycans are produced by both tumor cells and cells from the tumor stroma and can interact with growth factors, cytokines and integrins regulating their actions, thereby potentially contributing to tumor growth and progression [5-11].

Endocan, previously named Endothelial Specific Molecule-1 (ESM-1), a dermatan sulfate proteoglycan which is found freely circulating in the blood, is specifically secreted by endothelial cells and is preferentially expressed in lung and kidney tissues [12-16].

Recently, we have demonstrated that endocan has a tumorigenic activity. *In vivo*, 293 cells which express Endocan induce tumor formation when injected subcutaneously (s.c.) in SCID mice while they wild type counterparts do not. Similarly, endocan expression by tumorigenic HT29 adenocarcinoma cell line results in a markedly increase of the growth rate of the resulting tumors [17]. The tumorigenic properties of endocan are dependent on glycan chain but the role of the protein core, and more particularly of a Phenylalanine-rich (F-rich) region situated between residues F113 and F116, is also critical. Indeed, glycosylated endocan mutants with point mutations of the phenylalanine residues 115 and 116 failed to promote tumor growth in SCID mice [17]. Furthermore, endocan-mediated tumor formation was also inhibited by treatment with a specific blocking monoclonal antibody (mAb) targeted close to this region of the endocan protein core.

Endocan may be a key player in sustaining tumor growth and recent microarray analyses identified endocan, among other genes, as being one of the most significant molecular signatures defining a poor prognosis in breast [18], lung [19] but also in prostate cancers [20]. We already reported that endocan levels were markedly increased in the sera of patients with lung cancer and were related to tumor invasiveness [17,21].

The endocan gene was cloned by us in 1995. There is a single endocan gene localized on chromosome 5 (5q11.2) which spans 12 kb. The gene is organized into 3 exons separated by 2 introns. Exon 1 is encoded by 362 bp, exon 2 by 150 bp, and exon 3 by 1560 bp. Exon 1 and a part of exon 2 encode for an N-terminal cysteine-rich region of 110 amino acids in length. Exon 2 also encodes for the functionally-defined Phenylalanine-rich (F-rich) region (^113^FPFF^116^). Finally, exon 3 encodes for the C-terminal region that supports the O-glycanation site on serine 137. After deletion of the signal peptide, the mature endocan polypeptide is composed of 165 amino acids and carries only one GAG chain. Northern blot analysis identified initially only one mRNA product in human umbilical vein endothelial cells (HUVEC) [15]. However, RT-PCR has revealed an alternative spliced form of endocan mRNA with an internal deletion of 150 bp [22]. We also observed two distinct translated products of endocan in lysates from HUVEC suggesting the presence of products derived from alternative splicing [13]. Alternative splicing is a major mode of genetic regulation in higher eukaryotes and spliced variants or isoforms can have different or opposite physiological roles [23] and can be used as cancer biomarkers [24].

In this report, we analyze the tumorigenic effect of endocanΔ2, the alternatively spliced product of the endocan gene which lacks the sequence encoded by the exon two. Our data shows that the deletion of exon 2- related amino acid sequence impairs glycanation, endocanΔ2 being a "part time" proteoglycan. We also demonstrate that endocanΔ2 does not support tumorigenesis.

## Methods

### Cell culture

Endothelial cells, derived from umbilical vein (HUVEC) [25], were cultured on fibronectin-coated flasks in RPMI 1640 with 20 % FCS, 2 mM L-glutamine, 10 μg/ml heparin and 25 μg/ml endothelial cell growth supplement (Sigma). The 293 cells (ATCC CRL-1573), SV1 (derived from HUVECs by SV 40 transformation), and 293 Flp-In cells (Invitrogen) were cultured in DMEM with 10 % FCS and 2 mM L-glutamine; 100 μg/ml of Zeocin (Invitrogen) was added for the 293 Flp-In cells. The CHO (ATTC CCL-61) were cultured in α-MEM supplemented with 10 % FCS and 2 mM L-glutamine.

### RT-PCR and cDNA cloning

Two μg of total RNA isolated by TRIzol^® ^Reagent from HUVEC, and rat and mouse lungs, were reverse transcribed with M-MLV Reverse transcriptase (Invitrogen). Rhesus monkey lung cDNA was purchased from BioCat (Heidelberg, Germany). PCR was performed for 30 cycles with a 2 min hot start at 92°C, 1 min annealing at 55°C, and 30 s extension at 65°C with HotMaster Taq Polymerase (Eppendorf, Le Pecq, France). Endocan cDNAs were amplified with the following forward (S) and reverse (R) primers hybridizing in exon 1 and exon 3.

S1: 5'-ATGAAGAGCGTCTTGCTGCTG-3';

S2: 5'-ATGAAGAGCCTCTTGCTGCTG-3';

S3: 5'-ATGAAGAGCCTCTTGCTACTG-3';

R1: 5'-TCAGCGTGGATTTAACCATTT-3';

R2: 5'-TCAGCGCGGATTTAACCATTT-3';

Human and rhesus monkey endocan sequences were amplified with S1/R1; Mouse endocan with S2/R1; and rat endocan with S3/R2. For endocan and endocanΔ2 cloning, forward (5'-GAGGCAGCTGGGAAACATGAAG-3') and reverse (5'-GCCTTCTCTCAGAAATCACAG-3') primers were chosen to obtain complete reading frames. PCR products were subcloned in a pcDNA5/FRT vector (Invitrogen) and inserted into a pcDNA3.1 (+) vector (Invitrogen).

### Cell transfection

One μg of endocan-, endocanΔ2-, or control- pcDNA5/FRT or pcDNA3.1 (+) DNA was transfected using Lipofectamine 2000 (Life Technologies Inc., Cergy-Pontoise, France) into HEK 293 Flp-In or HEK 293 cells, as recommended by the manufacturer. The pcDNA5/FRT vector was co-transfected with the pOG44 vector which results in expression of the Flp recombinase. which mediates a homologous recombination event between the FRT sites from the HEK 293 Flp In cell genome and the pcDNA5/FRT vector. Stable transfected cells were selected using 100 μg/ml hygromycin B for HEK 293 Flp-In cells or 200 μg/ml G418 and cloned by limit dilution for HEK 293 cells. Endocan-pcDNA3.1 (+) was transfected into CHO-K1 cells under the same conditions.

### Endocan and endocanΔ2 immunoassays

Endocan and endocanΔ2 enzyme-linked immunosorbent assay (ELISA) were performed as previously described [13], using the in house anti-endocan MEP14 (IgG2a/K, capture) and MEC15 (IgG1/K, detection) monoclonal antibodies. Subsequent incubations with biotin-conjugated anti-mouse IgG1 (BD Biosciences- Pharmingen) and streptavidin-conjugated horseradish peroxidase (HRP) were followed by revelation with tetramethylbenzidine (Sigma).

### Immunoprecipitation and western blotting

Supernatants and cell lysate samples from 2–3 × 10^6 ^cells, were prepared as previously described [13]. The samples were run on 15% acrylamide gels and blotted onto nitrocellulose membranes (Hybond ECL, Amersham, Saclay, France). Detection was performed using MEP14 as the primary antibody probe, followed by affinity-purified, HRP-conjugated goat anti-mouse antibody (dilution 1:15,000) (Sigma) and an enhanced chemiluminescence (ECL) detection kit (Amersham).

### Analysis of endocan isoforms

The HEK 293 cell supernatant was passed through a 0.2 cm × 1.3 cm diethylaminoethyl (DEAE)-Sepharose column (BIO-RAD, Marne la coquette, France) run originally in 20 mM Tris HCl pH 7.4, 0.15 M NaCl. Bound endocan was eluted with 20 mM Tris HCl pH 7.4, 1 M NaCl, and concentrated in a 0.5 ml 30 kilo Daltons (kDa) molecular weight cut-off Vivaspin concentrator (Vivascience, Hannover, Germany). Samples treated with 1 unit/ml chondroitinase ABC (Sigma) overnight at 37°C and diluted 1:1 in sodium dodecylsulfate-polyacrylamide gel electrophoresis (SDS-PAGE) sample buffer without dithiothreitol (DTT) were subjected to PAGE and western blot analysis. Reduced conditions were obtained by addition of 0.1 M DTT in samples.

### Animal models

One day after receiving 100 μL of anti asialo-GM_1 _Ab (Wako Chemicals, Neuss, Germany) intraperitoneally, SCID mice were injected s.c. with 10^6 ^transfected HEK 293 or HEK 293 Flp-In cells resuspended in 200 μL DMEM without FCS. Presence of a palpable tumor was assessed once a week, and largest orthogonal diameters were measured using a Vernier caliper. Tumor volume was estimated using the formula: V = a*b^2^/2, where "a" is the largest and "b" the shortest diameter. Mice were euthanized when tumor volume reached at most 2 cm^3^. The protocol was approved by the local ethics committee and was compliant with the Directive 86/609/EEC on the protection of animals used for experimental and other scientific purposes.

### Tumor samples

We also collected tumor and normal lung samples from nine patients undergoing surgery for NSCLC. Immediately after surgical resection, the pathologist sampled both the tumor and the lung distant from the tumor. Each sample was immediately frozen at -20°C in TRIzol reagent (Invitrogen, Cergy Pontoise, France) for later RNA extraction. Both the protocol and the use of human tissues were approved by the local ethics committee, and the patients gave informed consent. Only patients with a clear histological classification as NSCLC, and without any previous treatment, were included in the study.

## Results

### Alternative splicing of the exon 2 of esm1 gene is restricted to primate species

**Figure 1:**
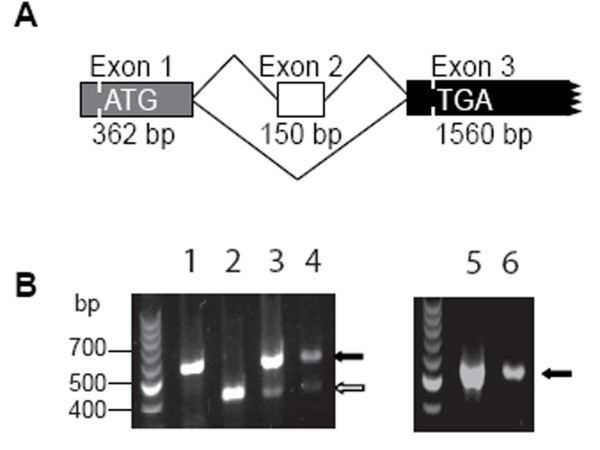
Expression of endocan and endocanΔ2 spliced products of *esm1 *gene. A: Scheme of the cloned human *esm1 *gene. (GenBank Accession Numbers AJ401091 and AJ401092). B: RT-PCR using primers hybridizing with sequences in exon 1 and 3 of *esm1*. Lane 1: endocan-HEK 293 cell line, lane 2: endocanΔ2-HEK 293 cell line, lane 3: HUVEC, lane 4: monkey lung, lane 5: rat lung, lane 6: mouse lung. The numbers to the left are molecular sizes in bp. Black arrows represent the endocan cDNA and white arrow represents endocanΔ2 cDNA.

Previous results on vascular endothelial growth factor (VEGF)- and fibroblast growth factor (FGF)-2- stimulated primary cultures of human capillary endothelial cells described two endocan-related mRNA species with 150 bp differences [22]. The 150 bp deletion corresponds to the deletion of the entire exon 2 (Fig. 1A) (GenBank Accession Numbers AJ401091 and AJ401092). We show here that the two alternative spliced products are also expressed in resting HUVEC (Fig. 1B). The new endocan mRNA species with the deletion of exon 2 was called endocanΔ2 (reported by us: GenBank Accession Number AJ973643). After cloning of the PCR product we observed that the reading frame was conserved and lead to the same stop codon. Endocan appeared to be more abundant than endocanΔ2. The expression of endocanΔ2 was then studied in mouse, rat and monkey lungs cDNAs. The expression of endocanΔ2 is observed in primate (human and monkey) but not in murine (mouse and rat) lungs (Fig. 1B).

**Figure 2:**
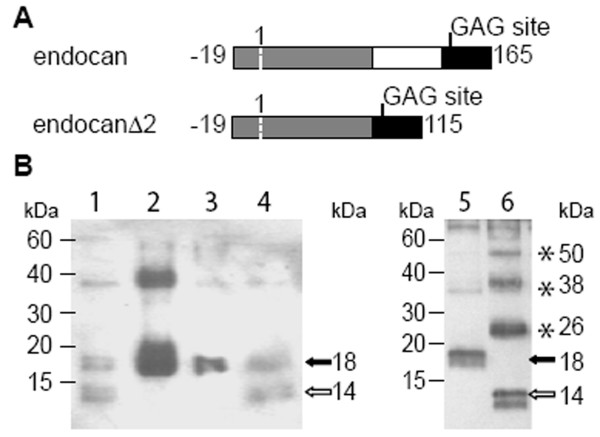
Endocan and endocanΔ2 translated products. A: Scheme of endocan translation products, deduced from cDNA sequences. Exon 1 is represented in grey, exon 2 in white and exon 3 in black. The first amino acid of the translated products is numbered -19, and the first of the mature translated products +1. B: Western Blot of cell lysates in non reducing conditions. Lane 1: HUVEC; lane 2: endocan-CHO-K1 cells; lanes 3 and 5: endocan-HEK 293 cells; lane 4: SV1 cells; lane 6: endocanΔ2-HEK 293 cells. Black arrow indicates the endocan translation product and white arrow the endocanΔ2 translation product. Stars indicate the multimeric forms of endocanΔ2.

The nucleotide sequence of endocan gene predicts that exon 1 encodes for the first 100 amino acids including the signal peptide, exon 2 encodes for 50 amino acids and exon 3 encodes for the last 34 amino acids. Thus, endocan and endocanΔ2 cDNA predicted translation products of 165 and 115 amino acids, respectively, with same N- and C-termini (Fig. 2A). This was consistent with immunoprecipitation studies from endothelial cell lysates, which revealed 2 bands at 18 and 14 kDa, the first band corresponding to endocan, the second band was suspected to correspond to the endocanΔ2 polypeptide (Fig. 2B lanes 1 and 4). This statement was further confirmed when HEK 293 cells transfected by endocanΔ2 cDNA generated specifically the 14 kDa band (Fig. 2B lane 6), but also other bands of 26, 38 and 50 kDa disappearing under reducing conditions. These results indicated that endocanΔ2 mRNA is primate specific, and is efficiently translated in HUVEC in a 14 kDa protein.

### Exon-2 derived sequence is needed for sustaining tumorigenesis

**Figure 3:**
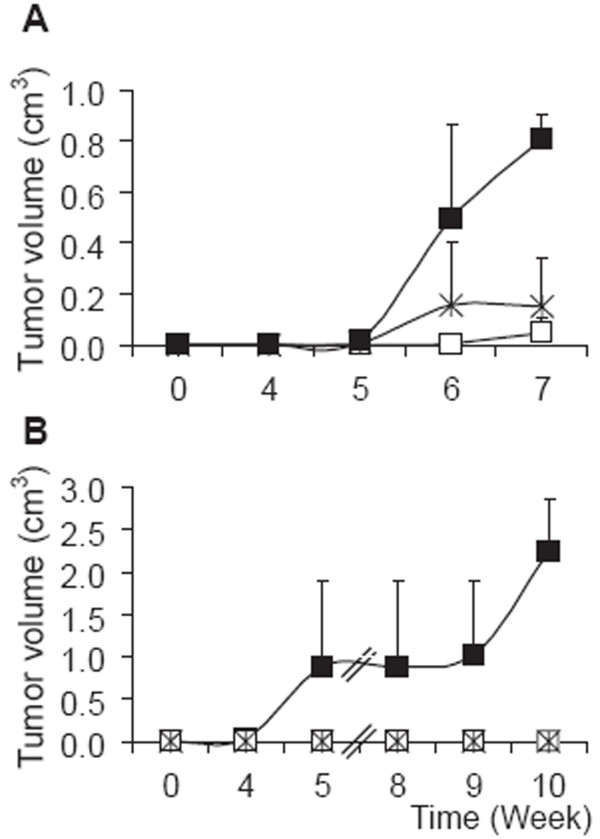
Kinetics of tumor growth. A: SCID mice were injected with 10^6 ^Flp-In HEK 293 cells overexpressing endocan (black box), endocanΔ2 (white box) or control vector (stars) (n = 8 animals per group). B: SCID mice were injected with 10^6 ^HEK 293 cells overexpressing endocan (black box), endocanΔ2 (white box) or control vector (stars) (n = 4 animals per group).

We have previously demonstrated that human endocan promotes tumor growth in a subcutaneous xenograft SCID-mouse model [17]. Here, we wondered if endocanΔ2 also exhibited such a tumorigenic activity. We established by homologous recombination HEK 293 Flp-In cells expressing endocan (22 ng/24 h/10^6 ^cells), endocanΔ2 (0.2 ng/24 h/10^6 ^cells), and then injected 10^6 ^cells into the skin of SCID mice. Every mouse that received endocan expressing HEK 293 Flp-In cells developed a tumor approaching 1 cm^3 ^in volume by 7 weeks (Fig. 3A). During the same time, mice receiving either control vector or endocanΔ2 expressing HEK 293 Flp-In cells developed smaller tumors measuring 0.15 cm^3 ^and 0.04 cm^3 ^respectively (Fig. 3A). Since the original experiments were performed with HEK 293 cell line and to discard a potential cell strain effect, a second series of experiments was performed using the HEK 293 cell line. All endocan-expressing HEK 293 cells (560 ng/24 h/10^6 ^cells) injected into SCID mice developed tumors by 5 weeks. After 10 weeks, the tumors grew up to 2 cm^3 ^(Fig. 3B). No tumors were observed in mice receiving endocanΔ2-HEK 293 cells (18 ng/24 h/10^6 ^cells) or the control vector-transfected cells (Fig. 3B). These data indicate that the expression of endocanΔ2 does not support tumor growth.

### Endocan Δ2 is not over expressed in lung tumors

We also investigated if the non- tumorigenic endocan Δ2 is overexpressed in human non small cell lung cancers (NSCLC). We use the same methodology as in our previous report [21]. In a series of 9 patients the median relative expression of endocanΔ2 to PECAM was 1.2 (interquartile range 1.1 to 1.4) while for the full length endocan it was 2.0 (interquartile range 1.4 to 2.4). Thus, we confirmed our previous results that there is a differential regulation of the alternatively spliced form endocan Δ2 in lung NSCLC [21].

### Deletion of exon 2 derived sequence impairs glycanation

**Figure 4:**
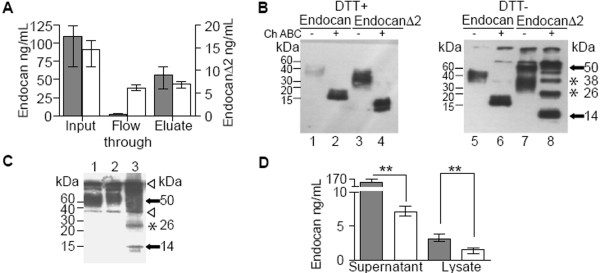
A: DEAE-sepharose binding property of endocan and endocanΔ2. The crude supernatants (Input) of transiently transfected HEK 293 cells with endocan (grey box) or endocanΔ2 (white box) (n = 4). The bound materials were eluted in 1 M NaCl (Eluate). Endocan and endocanΔ2 levels were measured by ELISA (median values with error bars showing the range). B: The eluates from DEAE-Sepharose column were digested overnight with chondroitinase (Ch) ABC. Digested (lanes 2, 4, 6, 8) and undigested (lanes 1, 3, 5, 7) samples were analyzed under reducing (lanes 1–4) or nonreducing (lanes 5–8) conditions. Black arrow indicates the endocan translation product and white arrow the endocanΔ2 translation product. Stars indicate the multimeric forms of endocanΔ2. C: Western blot analysis of endocan immunoprecipitated from endothelial or transfected cells supernatants. Lane 1: HUVEC; lane 2: endocan-HEK 293; lane 3: endocanΔ2-HEK 293. Black arrow indicates the endocan and endocanΔ2 translation products. Star indicate the multimeric form of endocanΔ2; Arrowheads indicate the non-specific bands originating from the secondary Ab. D. Endocan (grey box) and endocanΔ2 (white box) levels in the supernatant and cell lysate of HEK293 cells after 72 hours of culture assessed by ELISA (median, with error bars showing the range, n = 6) **, p = 0.022.

The glycan moiety of endocan is known to play important role in tumorigenesis [17]. We thus enquired if the glycanation of endocanΔ2 is modified compared to that of endocan. By its glycan, human endocan binds to the DEAE-Sepharose. The same property was observed for endocanΔ2 (Fig. 4A). The DEAE-bound endocanΔ2 was detected at 40 kDa in reducing conditions (Fig. 4B, lane 3). Treatment with chondroitinase ABC induced a shift from 40 to 14 kDa (Fig. 4B, lane 4), confirming that endocanΔ2, as well as endocan, is a proteoglycan with a chondroitin/dermatan sulfate chain. Surprisingly, not all endocanΔ2 is glycanated and 47.4 % (ranging from 42.9 % to 50.2 %) of endocanΔ2 was recovered in the flow-through of DEAE-Sepharose (Fig. 4A). This was confirmed using crude supernatants in non reducing conditions revealing two additional bands at 14 and 26 kDa below the glycanated endocanΔ2 spanning from 35 to 70 kDa (Fig. 4C, lane 3). Thus, the deletion of the exon 2-derived sequence impairs the glycanation of the endocanΔ2 polypeptide, partially explaining the lack of tumorigenic activity.

### Deletion of exon 2-derived sequence induces oligomerisation

The 14, 26 and 35–70 kDa forms of endocanΔ2 from crude supernatants suggested some conformational variants of endocanΔ2. First, the glycanated endocanΔ2 at 35–70 kDa (Fig. 4B, lane 7) resulted in a 40 kDa band under reducing conditions (Fig. 4B, lane 3). Second, chondroitinase ABC treated-endocanΔ2 revealed, other bands at approximately 26, 38 and 50 kDa (Fig. 4B, lane 8), in addition to the 14 kDa band, similarly to those observed in the lysates from endocanΔ2-HEK 293 cell (Fig. 2B, lane 6). The 14 kDa band is observed under reducing conditions only (Fig. 4B, lane 4). These results suggest that the presence of the exon 2-derived sequence results in a purely monomeric state for endocan, but in its absence oligomerization of endocanΔ2 is likely to occur, which may explain at least partially, the lack of tumorigenic activity of endocanΔ2.

### Deletion of exon 2-derived sequence alters protein expression

Despite identical cytomegalovirus promoter, signal peptide and N-terminal polypeptide of 81 amino acids, the respective secretion levels of endocan and endocanΔ2 were quite different. Transient transfection in HEK 293 cells resulted in 7.5 fold less secretion in cell supernatants, of endocanΔ2 than endocan (Fig. 4D). In addition, HEK 293 cell clones secreted 31 fold less endocanΔ2 than endocan. In cell lysates, levels of endocanΔ2 are also lower than for endocan (Fig. 4D), These findings were also true for other cell lines as HEK 293 Flp In or CHO.

## Discussion

Cancer is a leading cause of death in developed countries [26] and increasing efforts are directed towards understanding the biology of this disease. Endocan is an endothelial cell-derived proteoglycan which is overexpressed in various cancers [22,27,28]. *In vitro *as well as *in vivo *data demonstrated that endocan has a tumor growth promoting activity [17]. Moreover in human non small cell lung cancers endocan seems to reflect tumor angiogenic stimulation, is associated with tumor invasiveness and is related to patient prognosis. However endocan gene transcription results in two separate mRNA (endocan and endocanΔ2) and only the first one was completely characterized.

In this study, we have characterized the isoform of endocan, called endocanΔ2. This isoform was shown to have the same N- and C-termini as endocan, but was lacking the 50 amino acids encoded by exon 2. Our results show that this exon 2 sequence affects protein multimerisation, the glycanation status and the tumorigenic activity of endocan.

Initial Northern blots of both HUVEC and human lung mRNA have shown a unique endocan mRNA band, consistent with the unique 18 kD protein product from endocan transfected cells [15]. Subsequently, RT-PCR on microvascular endothelial cell lines revealed an alternative spliced form with a 150 bp difference [22]. We observed that in HUVEC lysates an additional endocan-related protein of 14 kD was present, which could be derived from either a specific peptide cleavage or an alternative splicing event. This last hypothesis is more plausible after our observation that two endocan mRNAs with a 150 bp difference were detected in HUVEC. The exon 2 of the human endocan gene is exactly 150 bp long, which definitively argues for an alternative splicing of human endocan gene resulting in a major form with all 3 exons, i.e. full-length endocan, and a minor form lacking exon 2, which we called endocanΔ2. This alternative splicing seems to be a primate-specific event. Despite the same genomic organization with three exons, only one mRNA, which contains all 3 exons, was observed in mice. We also show that the endocanΔ2 cDNA is translated into a 14 kD protein, thus demonstrating that the two bands of 18 and 14 kD in HUVEC represent the translation products of the two alternatively spliced forms of the endocan gene.

Alternative splicing is a major mode of regulation of the biological activities and the glycanation of proteins. One example is represented by CD44, which is a ubiquitous cell surface adhesion molecule involved in cell-cell and cell-matrix interactions. CD44 isoforms are derived from the differential utilization of 10 variable exons (CD44v1-v10). Variations in the splicing process occur during tumor progression and may play a major role in tumorigenesis [29]. Alternative splicing can also change the glycosylation status of a proteoglycan. For example, in addition to the large diversity of protein isoforms resulting from alternative splicing, CD44 can also undergo different post-transcriptional modifications such as N-glycosylation and O-glycosylation. Many forms of CD44 are glycanated by chondroitin sulfate, however the v3 exon directs glycanation with heparan sulfate. Here we demonstrated that endocanΔ2 is secreted mostly as a chondroitin/dermatan sulfate proteoglycan like endocan, so the loss of exon 2 does not change the specificity of glycanation. Surprisingly, we identified endocanΔ2 as a "part-time" proteoglycan, based on the presence in the cell supernatants of both non-glycanated and glycanated forms. The respective tumor-inducing effects of endocan and endocanΔ2 have been examined in a tumor xenograft mouse model. The tumorigenic effect of endocan was clearly confirmed, independently of both the vector and the subtype of 293 cells used. In contrast, endocanΔ2 does not promote tumor growth. This result could be explained by (i) the absence of the exon-2-containing F-rich region, which is needed for tumorigenesis [17] (ii) the partial lack of the glycan chain previously shown to play an important role in the tumor promoting activity of endocan [17], and (iii) the formation of distinct disulfide-linked oligomers by endocanΔ2, which might also affect the pro-tumoral activity by substantial modifications of protein conformation. Indeed, the deletion of 6 out of the 18 cysteines present in endocan, as a result of the splicing out of exon 2, may result in a reorganization of the disulfide bonds that alter the balance between mono- and oligo-merization. Some of these changes may also be inter-related in that a tendency towards protein multimerisation may restrict glycanation, or *vice versa*. The low levels of protein secretion for endocanΔ2 could eventually explain the lack of its tumorigenic activity. However very low levels of endocan secretion are necessary to sustain tumor growth as demonstrated by the tumorigenicity of 293 Flp-In cells which secrete very low levels of endocan. The secreted levels of endocan Δ2 are higher than the spontaneous secretion of endocan by the 293 Flp-In cells and thus do not sustain this hypothesis. Whatever the mechanism(s), these results nevertheless strongly suggest a key role for the exon 2-derived sequence in the pro-tumorigenic effect of endocan.

Recently, many microarray and substractive hybridization analyses of the gene expression profiles in several human cancers have identified endocan as being one of the genes defining a tumor signature, associated with poor prognosis with an increased risk of death or invasive disease in melanoma, breast, kidney prostate and lung cancers, [18-20,28,30]. Interestingly, in human lung cancer, we suggested previously and confirmed in the present paper that the mRNA of endocan is increased in tumor endothelial cells, while the mRNA of endocanΔ2 remained unchanged, indicating a putative regulation of the splicing of the endocan gene [21]. In parallel, we have found elevated levels of endocan circulating in the blood [21]. Several lines of evidence suggest that endocan expression normally predominates over endocanΔ2 expression *in vivo*. Firstly, the expression of endocanΔ2 mRNA is lower than that of endocan mRNA in HUVEC and in human lung tissues; secondly, the non-glycanated endocanΔ2 observed in endocanΔ2–293 cells, was not detected in immunoprecipitates from HUVEC supernatants; and thirdly, treatment with chondroitinase ABC did not reveal the endocanΔ2 protein core in either HUVEC supernatants or in normal human serum.

## Conclusion

Taken all together, these results show that the alternative splicing of endocan gene may be one important mechanism that might downregulate the function of endocan. The presence of an isoform which lacks the tumor promoting effect of endocan implies that in subsequent molecular biology analyses (microaray or real time PCR) the choice of the primers should be done very carefully in order to distinguish only the tumorigenic form. Proteomic analyses should also take into account these differences if they are done from crude tissues extracts. This does not apply to the quantification of the circulating form of endocan. Even if the available ELISA systems detects both endocan isoforms, the endocanΔ2 is poorly secreted and accounts only for a very small fraction of the secreted protein.

In conclusion, we characterized a novel alternatively spliced product of the endocan gene that is devoided of tumorigenic activity. Our data strongly suggest that endocan exon 2-derived sequence may represent a potential therapeutic target in human lung cancer.

## Competing interests

The author(s) declare that they have no competing interests.

## Authors' contributions

FD, BDG participated in the design of the study, performing experiments analysis and interpretation of data; PhL, AS, MD, PhG were involved in the design of the study and analysis of data; all authors were involved in drafting and revising the manuscript for important intellectual content and have given final approval of the version to be published.

## Pre-publication history

The pre-publication history for this paper can be accessed here:


